# Supplementary material for: Chromothripsis during telomere crisis is independent of NHEJ, and consistent with a replicative origin
Source: Genome Res. 2019 May;29(5):737–49. doi: 10.1101/gr.240705.118 (PMC6499312; doi:10.1101/gr.240705.118)
Supplement: Supplemental Material [file supp_gr.240705.118_Supplemental_file_1.zip › contigs/annotated_contigs/DB51/contig.3.DB51_length_681_mean_cov_5.43465491924.docx]

**DB51_length_681_mean_cov_5.43465491924**

```
TAGACAGGACAACATCTGAATTTTGGAACTACAATATCTTACATGATGCTTTGCTCAAACTAGCTCTCAATAATTTTATTTATTCACAT
 >chr6:117020747-117021075 - E=1e-182
TCAATCATTTCTTCATTCATTCAATTGGAATTAAAAAGCCAAATAATAGGTATCAAGAGGTCTGGGTTTTAGTCTGGACTTAGGGCTTA

TATAAAGGAATGGAACCAGATTTCATGATCTCTAGTTCAAAATAATTGACAATAAAACTGTAACATGACTAAAGTAGCCTCTTAGAGAC

CCTTATTGTAAACATTCTCAACAATTTTTCACATTCACACATGCAATACATTCCTTTAC|TT|TTTCTTACTTTCTCTCTCTCTTTCTC
                                                            >chr18:68644562-68644607 -
TCACTTTCTCTCTCTC|T|ACAGTGGTAAAGGGCCATACATCAAGAAAACATAAAAGTCATAAATGCGTATGCATTTATACACTGTAAA
E=2e-15          >chr18:68501778-68502089 - E=2e-175
CAACAGTCCTACAAAATATGTGAAGCAAAAACTGATCAACTGAAATGAGAAATAACAGACAAACACCACAGTTATAGTGAAGGCTTCAA

GACTCATCTTTCACAAAATTGAAGAGAAAATCTAGAAGGATAAAGAACTCAACAATATTGTCAACCAAAAAGATCAAATCAACATCCAT

AAAACAATTCACTCAACCACAACAAAATATATATGCTTTCTTTATACTTCCACAAAATACGT
```
